# Evaluating the adoption of evidence-based practice using Rogers’s diffusion of innovation theory: a model testing study

**DOI:** 10.15171/hpp.2018.03

**Published:** 2018-01-07

**Authors:** Mohammad Mehdi Mohammadi, Roghayeh Poursaberi, Mohammad Reza Salahshoor

**Affiliations:** ^1^Students Research Committee, School of Nursing and Midwifery, Kermanshah University of Medical Sciences, Kermanshah, Iran; ^2^Department of Education, Payame Noor University (PNU), Iran; ^3^Medical School, Kermanshah University of Medical Sciences, Kermanshah, Iran

**Keywords:** Diffusion of Innovation, Evidence-Based Practice, Nursing Students

## Abstract

**Background:** Despite the emergence and development of evidence-based practice (EBP) in recent years, its adoption continues to be limited. This study used Rogers’s diffusion of innovation theory to identify the factors that advance EBP adoption, determine the process by which such adoption occurs, and develop an EBP adoption model.

**Methods:** This descriptive correlational study with model testing design conducted in 2015.Data were collected from 482 individuals (322 nurses and 160 nursing students) applying a demographic information questionnaire, a standard scale for the perception EBP attributes, an EBP scale, and an individual innovation inventory. The relationships between variables we reexamined by path analysis.

**Results:** The results showed that EBP adoption had a significant positive relationship with individual innovation (r = 0.578, P < 0.001), knowledge (r = 0.657, P < 0.001), attitude (r = 0.623,P < 0.001), and age (r = 0.357, P < 0.001). The findings of path analysis indicated that the goodness of fit indices such as goodness of fit index (GFI) = 0.999, comparative fit index (CFI)= 0.999, root mean square error of approximation (RMSEA) = 0.036 were in the ideal ranges.Knowledge (total effect=0.309, P < 0.001), attitude (total effect = 0.372, P = 0.002), and work experience (total effect=0.321, P = 0.003) had the highest coefficient in the model.

**Conclusion:** The results suggested that EBP adoption was influenced by various factors, such as individual innovation, attitude, knowledge, and the perception of EBP attributes. Among these factors, attitude had the greatest effect on EBP adoption. The findings can serve as a guide for the identification of factors that effectively influence EBP adoption. They can also be used as bases for the design of training programs intended to enhance the adoption of EBP.

## Introduction

Evidence-based practice (EBP), which was first introduced by Rhazes and Avicenna,^1^ can be defined as the application of the best research findings on clinical decision making.^2^ Despite the emergence and development of EBP in recent years, clinical practitioners have adopted the concept only to a limited degree, failing to provide EBP-conformant health care services to about 30% to 40% of patients in the United States. The non-application of EBP is the starting point of a gap between “what is there and what will be best.”^3^ EBP adoption is important because its objectification in clinical decisions is expected to reduce the effects of subjective errors, the use of obsolete information, and the exercise of practices based on unsubstantiated experiences.^4^

Nurses constitute a massive group of practitioners responsible for providing clinical services, but ongoing advances in medical sciences point to the reality that the effectiveness of a nurse’s knowledge and the lifespan of information are short-lived and that previously learned content increasingly become obsolete.^5^ These problems can be addressed through the use of EBP, which not only helps health care providers make the right decisions but also eliminates the effects of outdated science; it opens a window to modernity and the enjoyment of a secure practice for health care providers.^5^ EBP adoption necessitates behavioral changes among health care providers—a goal that was indicated as possible in a systematic review of studies on the concept; health care practitioners have the potential to further improve their provision of care by applying a reliable framework, such as evidence-based care.^6^

In Iran, the health sector is characterized by limited EBP use and a lack of studies on various EBP-oriented training programs.^7,8^ A systematic review of the country’s situation showed that health care providers exhibit low awareness of specialized EBP terminology.^8^ Some other international studies, however, reported that the state of knowledge and awareness regarding EBP and its application in Iran is at a desirable level.^9-11^ Notwithstanding mixed findings, the narrow scope of EBP adoption in Iran is an undeniable issue. Because transition to EBP implementation is expected, a favorable strategy is for health care providers to adopt the practice beginning at the first stage of transition as the spread of EBP depends on early adoption.^12^ This goal can be achieved on the basis of Rogers’s diffusion of innovation theory, which serves as a conceptual framework for the identification of conditions that advance innovation adoption and related methods of adoption.^13,14^ The theory enables the examination of how certain clinical behaviors are adopted and allows focus to be directed toward perceived innovation attributes that increasingly drive adoption.^15^ Rogers considered the attributes of an innovation to be effective factors for adoption; he stated that the five attributes of an innovation—namely, relative advantage, compatibility, simplicity, observability, and trialability—are determinants of the adoption and diffusion of the innovation in a target clinical community.^15^ The author also believed that the diffusion of innovation theory provides all the steps necessary to promote the adoption of a new idea and that individuals’ perception of an innovation’s features can predict adoption.^14^

**Figure 1:**
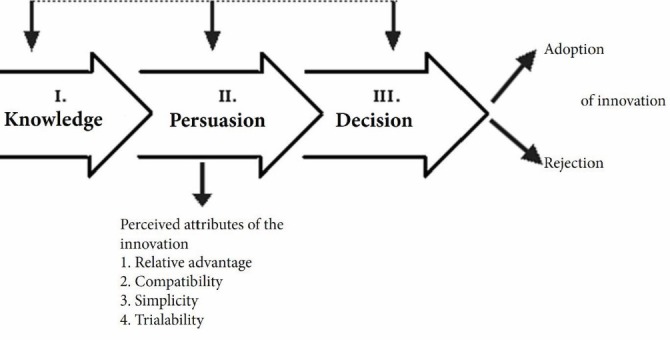
The process of adoption in Rogers’s diffusion of innovation model.^17^

According to the Rogers’s diffusion of innovation model (Figure 1), *Knowledge* is produced when an individual is exposed to an existing innovation and acquires some understanding about its mechanisms and functions. To reach the *Persuasion* stage, the individual must form a view toward the innovation based on its perceived attributes (relative advantage, complexity, and so on). For *Decision* to occur, the individual must be involved in an activity that would ultimately require him or her to make a choice between using or dismissing the innovation. For *Adoption* to occur, the individual must arrive at the decision that the innovation is the best available option for moving forward.^16^

Correspondingly, this study used the diffusion of innovation theory to identify the factors that promote EBP adoption and determine the process by which the concept is adopted by the nursing students of the Nursing and Midwifery Department at Kermanshah University of Medical Sciences and the nurses working at Kermanshah Educational Hospitals. The theory was also employed in developing an EBP adoption model. In this work, the innovation examined was EBP.

## Material and Methods

### Study design and participants 

This correlational study was conducted in 2015 and involved 482 individuals (322 nurses and 160 nursing students). The final sample of nurses was selected from a total of 1950 individuals using random sampling and the Krejcie and Morgan table.^18^ All of the participating nursing students were selected via census. The inclusion criteria for the nursing students was being at the third and fourth years of study (fifth to eighth semester). The inclusion criteria for the nurses was at least one year’s work experience in the hospitals. The exclusion criteria were unwillingness to continue participation and faulty completion of questionnaires.

**Figure 2:**
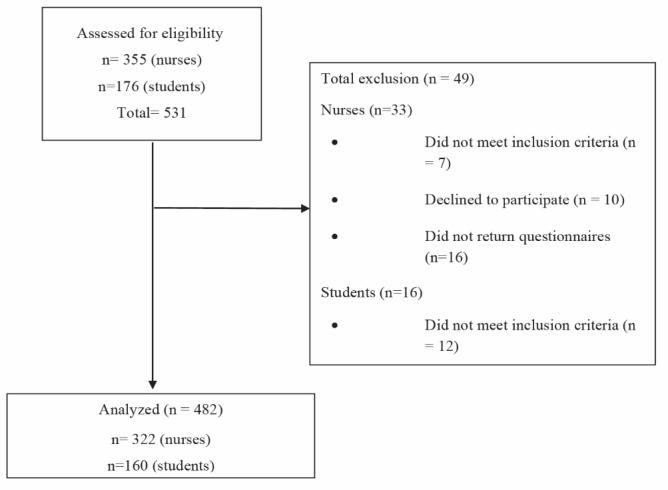
The flow chart diagram of the study.

At the time of the study, 176 nursing students were at their third and fourth years at the university; 16 of them did not meet the inclusion criteria or refused to participate. The remaining 160 adolescents were recruited. About the nurses, 355 nurses were assessed and 33 of them were excluded from the study for some reasons (Figure 2).

### Research instruments

Four instruments were used to collect data:

*Demographic information checklist:* This checklist consists was about the individual characteristics (age [year], gender [male/female], grade point average, and work experience [year]) of the participants.

*Standard scale for the perception of EBP attributes:* This questionnaire consists of 25 items that are scored using a 5-point Likert scale ranging from 1 (“strongly disagree”) to 5 (“strongly agree”). The total score that can be obtained by summing the items scores (ranging from 25 to 125). The higher the score on each of the EBP attributes, the greater the perception of that attribute. This questionnaire and its corresponding psychometric analysis were first designed and evaluated, respectively, by Moore and Benbasat.^19^

It was then standardized for application in the context of Iran by Ashktorab et al. In this regard, the content validity of the questionnaire was assured by experts (content validity index [CVI] = 0.98), in order to confirm internal consistency and stability, Cronbach alpha and interclass correlation coefficient (ICC) were used (α = 0.70 and ICC = 0.78).^20^

The questionnaire covers Rogers’s five innovation attributes, which are explained as follows:

- Relative advantage: This attribute refers to the benefits that EBP use presents to the process of nursing tasks, the improvement of the quality of clinical care, and the effectiveness of nursing services. It also pertains to the costs, benefits, and potential advantages that an innovation carries (EBP in this work).
- Compatibility: This attribute refers to the match, consistency, and appropriateness of EBP use for nursing work; in other words, compatibility is a perceived attribute of EBP that is consistent with the existing values, past experiences, and potential need associated with further innovation adoption.
- Simplicity: This attribute refers to the ease of EBP application for an individual.
- Observability: This attribute pertains to how observable the effects and outcomes of EBP adoption are to users and other individuals.
- Trialability: Trialability revolves around the extent to which EBP is trialable for a nurse; note that trialability is of a limited basis.^15^

Note that the range of variations in score for each of the attributes is 5 to 25.

*EBP scale:* This questionnaire was designed and evaluated by Rubin and Parrish.^21^ It consists of 34 items under three subscales: knowledge (10 items), attitude (14 items), and adoption (10 items). The items are scored using a 5-point Likert scale ranging from 1 (“strongly disagree”) to 5 (“strongly agree”). Scores range from 10 to 50 in the knowledge subscale, from 14 to 60 in the attitude subscale, and from 10 to 50 in the adoption subscale.^21^ The higher the score in each subscale, the higher the degree of that subscale. The content validity of the questionnaire for the Iranian context were assessed by experts, with the evaluation registering acceptable face and content validity (CVI = 0.98). Cronbach alpha and ICC were used to confirm internal consistency and stability, respectively. The values of the subscales in this regard were acceptable: knowledge (α = 0.82 and ICC = 0.94), attitude (α = 0.80 and ICC = 0.94), and adoption (α = 0.75 and ICC = 0.74).^22^

*Individual innovation inventory:* This questionnaire was first designed and validated by Kleysen and Street.^23^ It comprises 14 questions, for which items are scored using a 5-point Likert scale ranging from 1 (“strongly disagree”) to 5 (“strongly agree”). The score obtained in the instrument ranges from 14 to 60, and a high score indicates that a respondent exhibits strong innovative talent.^23^ The validity and reliability (content and face validity) of the questionnaire for the context of Iran was evaluated and confirmed by experts (CVI = 0.98); the instrument’s reliability was also found to be acceptable (α = 0.87; ICC = 0.91).^22^ In the present study, the reliability of the questionnaire was confirmed (α = 0.83; ICC = 0.87).

### Data collection 

For the data collection from the nurses, the Kermanshah educational hospitals were visited and the permit for data collection was presented to the nursing office of the relevant hospital, the researchers obtained information on the number of nurses working in each ward, and constructed a sampling framework for each ward. In proportion with the number of nurses working in each of the wards, participants were recruited via randomized stratified sampling. Given that the nurses generally work in the morning shifts and have no opportunity to complete the questionnaires, the instruments were delivered to the nurses, and they were asked to complete them at their own pace. The questionnaires were returned by the nurses the following day.

For the data collection from the nursing students, the Nursing and Midwifery Department was visited and the permit for data collection was presented. The researchers obtained the list of students who were undergoing internship at their third and fourth years at the university, after which we sampled participants through the census method. The students were then asked to complete and submit the questionnaires.

### Data analysis

The data were analyzed using SPSS (version 22.0; SPSS Inc., Chicago, IL, USA) and AMOS (version 22.0, Smallwaters Corporation, Chicago, IL, USA). Normality was assessed using the Kolmogorov–Smirnov test, which revealed that the data distribution was normal. Then, an independent *t* test was run to compare the 2 groups of respondents. Pearson correlation coefficient was used to determine the correlation between each of the variables and EBP adoption. A path analysis was performed using AMOS version 22.

In order to conduct the path analysis, 5 steps of the modeling process were carried out‏.

*Step 1: Model Specification*

In this regard, the Rogers’s diffusion of innovation model was used as a conceptual model. Rogers introduced the variables of *knowledge* and *perceived EBP attributes* as factors influencing the EBP adoption. On the other hand, variables such as individual innovation, knowledge and experience have also been introduced in some studies as factors influencing the EBP adoption.^24^

*Step 2: Model Identification*

In this stage, the *order condition* was considered. For this purpose, the following relationship must be existed:

The number of distinct values in the sample variance–covariance matrix must be greater than or equal the number of free parameters to be estimated. In present study, we have the following free parameter values: 3 correlations among the independent variables, 3 independent variable variances, 11 path coefficients and 3 equation error variances.

Therefore, the number of free parameters in the present study was 20, In order to calculate the number of distinct values, the following equation was used (*p* is the number of observed variables).

[*p* (*p* + 1)]/2 = [6(6 + 1)]/2 = 21

Therefore, the number of distinct values (21) is greater than the number of free parameters (20) and the *order condition* was met.

*Step 3: Model Estimation*

In model estimation, using the AMOS software, the maximum likelihood estimation method was applied to estimate the parameter.

*Step 4: Model Testing*

In this step, goodness of fit indexes reported the fitness of the model and they were in the ideal range (Table 1).

*Step 5: Model Modification*

In present study, the ideal range of goodness of fit indexes of model were met; therefore, modification for better fitness was not required.

## Results

As previously stated, 482 individuals (nurses = 322 or 66.8%, nursing students = 160 or 33.2%) participated in the research. The mean age of all the participants was 30.82 (SD: 7.66) years; the mean age of the nurses was 31.79 (SD: 7.55) years, whereas that of the nursing students was 28.87 ± 7.51 years. The mean and standard deviation of the rate of EBP adoption were 31.65 (SD 7.33) and 20.26 (SD: 4.47) among the nurses and nursing students, respectively, indicating a significant difference between the groups (*P* < 0.001). The overall mean values of perceived EBP attributes were 71.38 (SD; 23.11) and 56.24 (SD; 9.35) for the nurses and nursing students, respectively, showing a significant difference between the groups (*P* < 0.001). The mean and standard deviation of each of the perceived EBP attributes for the nurses and nursing students are shown in Table 2.

As shown in Table 3, EBP adoption was significantly positively related to knowledge, attitude, individual innovation, age, relative advantage, simplicity, observability, and trialability in both groups.

The results on the effects of each of the variables of the path analysis model on EBP adoption are presented in Table 4. Knowledge, attitude, and work experience provided the highest contribution to the prediction of EBP adoption.

**Figure 3:**
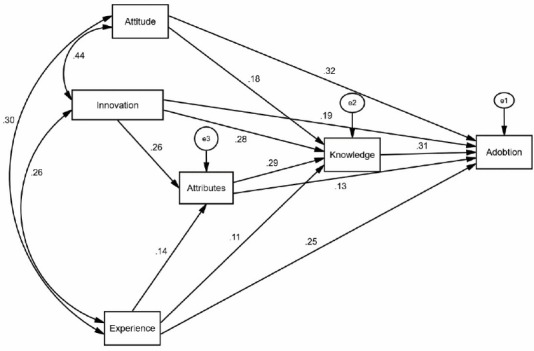
Novel EBP-oriented version of Rogers’s model.

Figure 3 demonstrates the standardized path coefficients. The results reported that all paths were significant at *P* < 0.05.

## Discussion

This study developed a model of EBP adoption with Rogers’s diffusion of innovation theory as the conceptual framework. The theory maintains that the perception of an innovation’s attributes can predict the adoption of that innovation. In this research, the perceived attributes of EBP predicted the adoption of the practice. Among the attributes studied in this research, relative advantage, simplicity, observability, and trialability had a significant positive correlation with EBP adoption. The positive correlation between relative advantage and EBP adoption indicated that EBP adoption increases with increasing perception of advantages such as the cost-effectiveness, potential benefits, and effects of EBP on the promotion of clinical care. This finding is consistent with that derived by Ashktorab et al.^20^ It also highlighted the need for emphasis on the benefits of EBP in training interventions. In designing a training program for the improvement of EBP adoption, part of the program should be allocated to training regarding the advantages of the practice.

Simplicity was positively correlated with EBP adoption, indicating that the ease of use and simplicity perceived by a community that wants to adopt EDP hastens adoption.^15^ This finding underscores the need to simplify training interventions as this would enhance EBP adoption. Put differently, EBP should be explained in plain language in a training program and should not be depicted as a complex process.

Observability was positively correlated with EBP adoption. EBP’s effects and outcomes and their positive correlation with EBP adoption indicates that as the positive outcomes and effects of EBP become more observable and tangible to nurses, its adoption rate increases. Consistent with the present study, Ashktorab et al regarded observability as one of the attributes of an innovation; the authors reported that as the perception of community members of this attribute rises, the adoption of the innovation increases.^20^ Emphasizing observability in EBP training is therefore important. The correct administration of EBP training is expected to enhance the tangibility of clinical services and enable caregiving personnel to experience improvements to service quality. We further recommend that the effectiveness of EBP training be improved by inviting successful and pioneering individuals in the use of EBP to administer training. This strategy will enable others to perceive the results of using EBP more tangibly.

Trialability was correlated with EBP, indicating that as nurses’ perception of EBP trials and trialability increase, EBP adoption also rises.^15^ The path analysis results also showed that health care providers’ individual innovation, work experience, and attitudes are factors that effectively influence EBP adoption. Work experience, the perception of attributes, and individual innovation can indirectly influence EBP because of their effects on level of knowledge and the direct effects of EBP adoption. This finding is consistent with the model that Pashaeypoor et al established.^24^ The interesting and striking point about this finding is that the diffusion of innovation theory emphasizes individuals’ past status and the existence of innovation as important in the acquisition of knowledge about innovation. Rogers stated that innovation is an idea or a practice that requires new knowledge and/or the development of a new opinion about an idea or practice. He regarded knowledge as the first step in innovation adoption as this is the avenue through which an individual develops awareness of innovation.^25^ In the present study, individual innovation, the perception of attributes, and work experience can indirectly influence the knowledge of an EBP adopter and directly affect EBP adoption. What is clear in our model is that knowledge is the central mediator. Consistent with the current research, Albarrak et al discussed the importance of knowledge in EBP adoption ^26^. Therefore, increased attention should be paid to augmenting individual knowledge about EBP.

Individual attitudes toward EBP also influenced EBP adoption. The model indicated that attitude exerted the strongest effect on EBP adoption. In his PhD dissertation, Palmar demonstrated that attitudes can predict EBP adoption.^27^ What assumes importance in this regard is that interventions and programs designed to promote EBP adoption should drive transition to EBP through the use of cognitive principles that can change people’s attitudes in positive ways.

This study contributes to the literature through its verification of Rogers’s diffusion of innovation model and through our incorporation of additional elements (work experience, attitude, etc) into the framework. We also discovered the relationships between these elements and established a new model that can serve as a novel EBP-oriented version of Rogers’s model. Researchers can use the results of this research to expand the proposed model.

A limitation of the study was the fact that the nurses lacked time to complete the questionnaires. They were simply asked to fill out the instruments at the right time and return the questionnaires the following day. Other limitation of this study was the cross-sectional nature of the study and causation cannot be inferred.

## Conclusion

This study examined EBP adoption as an innovation on the basis of Rogers’s diffusion of innovation theory. The results showed that EBP adoption was influenced by various factors, such as individual innovation, attitude, knowledge, work experience, and perception of EBP attributes. Attitudinal promotion exerted the greatest effect on EBP adoption, and this effect occurred as a direct and non-interactive impact. Individual innovation, the perception of attributes, and work experience influenced EBP adoption both directly and indirectly because of their effect on knowledge. The findings can facilitate the process of EBP adoption by health care providers and thereby lead to health promotion in general. In the future studies, it is suggested that, the researchers consider each of the influential factors introduced in this work in the design of training programs intended to improve EBP adoption.

## Ethical approval

This study was conducted under the supervision and with the approval of Kermanshah University of Medical Sciences (Grant No. 94270). As the research objectives were explained to the participants, they were assured that their information will be kept completely confidential. Each participant was also informed of his/her right to withdraw from participation in the research.

## Competing interests

There are no conflicts of interest.

## Authors’ contributions

MMM and RP designed the study. MMM and MRS collected the data and performed study supervision. RP analyzed data. MMM and RP and MRS contributed to drafting the manuscript‏.

## Acknowledgements

The authors gratefully acknowledge the Research Council of Kermanshah University of Medical Sciences for the financial support. All nurses and nursing students who sincerely helped us in this project are hereby thanked.

**Table 1:** Goodness of fit indexes of the model

**Goodness of fit indexes**	**Ideal range**	**Acceptable range**	**This study**
GFI	0.95 ≤GFI ≤1	0.9 ≤ GFI <0.95	0.999
AGFI	0.95 ≤AGFI ≤1	0.9 ≤ AGFI <0.95	0.976
NFI	0.95 ≤NFI ≤1	0.9 ≤NFI <0.95	0.998
CFI	0.95 ≤CFI ≤1	0.9 ≤CFI <0.95	0.999
χ2/*df*	χ2/*df* ≤2	χ2/*df* ≤5	1.621
RMSEA	0 ≤RMSEA <0.05	0.05≤RMSEA<0.08	0.036, 90% CI = 0.001-0.133

Abbreviations: GFI, goodness of fit indices; AGFI, adjusted goodness of fit index; NFI, normed fit index; CFI, comparative fit index; RMSEA, root mean square error of approximation; CI, confidence interval; *df*, degrees of freedom.

**Table 2:** Mean and standard deviation of perceived EBP attributes among nursing and nursing students^a^

**Perceived EBP attributes**	**Nurses**	**Nursing students**	***P*** ** value** ^b^
Relative advantage	14.95±6.33	9.08±2.17	0.001
Compatibility	13.92±7.21	11.15±3.19	0.001
Simplicity	13.16±5.86	11.19±2.49	0.001
Observability	15.69±6.12	13.91±3.09	0.002
Trialability	13.68±5.65	10.91±2.89	0.001

Abbreviation: EBP, evidence-based practice. ^a^All data are presented as mean ± SD. ^b^The independent samples *t* test was used.

**Table 3:** Correlation between perceived EBP attributes and other factors related to EBP adoption

**Variable**	**EBP adoption**					
	**Nurses**		**Nursing students**		**Nurses + Students**	
	**Correlation coefficient**	***P *** **value**	**Correlation coefficient**	***P *** **value**	**Correlation coefficient**	***P *** **value**
Relative advantage	0.149	0.008	0.641	<0.001	0.315	<0.001
Compatibility	0.051	0.361	0.183	0.021	0.053	0.244
Simplification	0.069	0.221	0.023	0.771	0.161	<0.001
Observability	0.187	<0.001	0.131	0.102	0.211	<0.001
Trialability	0.257	<0.001	0.198	0.012	0.309	<0.001
Individual innovation	0.566	<0.001	0.394	<0.001	0.578	<0.001
Knowledge	0.545	<0.001	0.641	<0.001	0.657	<0.001
Attitude	0.775	<0.001	0.183	0.021	0.623	<0.001
Age	0.281	<0.001	0.249	0.004	0.357	<0.001

Abbreviation: EBP, evidence-based practice.

**Table 4:** Direct, indirect and total standardized effect of predictive variables on EBP adoption

**Variable**	**Effect type**								
	**Direct effect**			**Indirect effect**			**Total effect**		
	**Estimate**	**95% CI**	***P*** ** value**	**Estimate**	**95% CI**	***P*** ** value**	**Estimate**	**95% CI**	***P*** ** value**
Perceived EBP attributes	0.134	0.030-0.073	<0.001	0.091	0.024-0.046	0.002	0.225	0.065-0.112	0.001
Individual innovation	0.187	0.062-0.121	<0.001	0.148	0.059-0.089	0.002	0.332	0.138-0.187	0.003
Knowledge	0.309	0.401-0.602	<0.001	-	**-**	**-**	0.309	0.401-0.602	<0.001
Attitude	0.318	0.212- 0.306	<0.001	0.054	0.031-0.062	0.001	0.372	0.232-0.370	0.002
Work experience	0.253	0.238-0.366	<0.001	0.067	0.061-0.109	0.001	0.320	0.303-0.457	0.003

Abbreviations: CI, confidence interval; EBP, evidence-based practice.
